# Congenital Left Coronary Atresia: Diagnostic Pearls and Management Challenges in Two Children Presenting with Syncope

**DOI:** 10.1016/j.case.2021.05.003

**Published:** 2021-06-25

**Authors:** Rachel T. Sullivan, Jennifer F. Gerardin, Peter C. Frommelt, Todd M. Gudausky

**Affiliations:** Division of Pediatric Cardiology, Medical College of Wisconsin, Milwaukee, Wisconsin

**Keywords:** Congenital coronary anomaly, Coronary ostial atresia, Echocardiography, Coronary angiography, Pediatric cardiac surgery

## Abstract

• LMCA ostial atresia has variable clinical presentation.
• Diagnosis of LMCA ostial atresia is challenging and often requires multiple modalities.
• Coronary revascularization is possible for LMCA ostial atresia by varying surgical techniques.

## Graphical abstract

**Figure:**
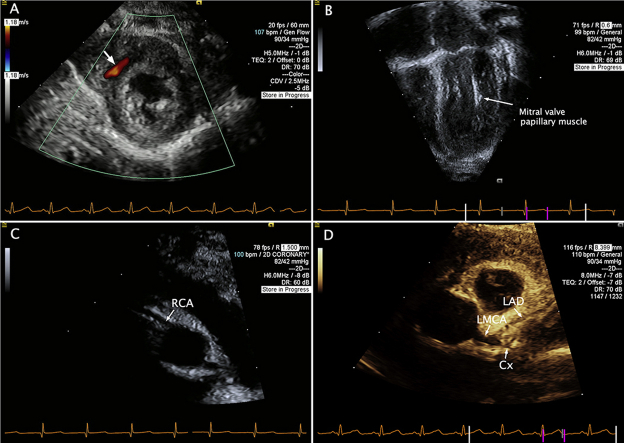

## Introduction

Congenital atresia of the left main coronary artery (LMCA) is a rare anomaly in which the LMCA does not receive antegrade flow from any vessel and instead receives exclusively retrograde flow from right coronary artery (RCA) collaterals. Its estimated incidence is 0.04%.^1^ Clinical presentation is varied and can manifest as heart failure and sudden cardiac death in infancy and childhood or as angina in adulthood.^2^ We present two pediatric cases with different clinical presentation, subtle initial diagnostic findings, and surgical management challenges. This case series received proper ethical oversight, including consent from patient guardians.

## Case 1 Presentation

**Figure 1:**
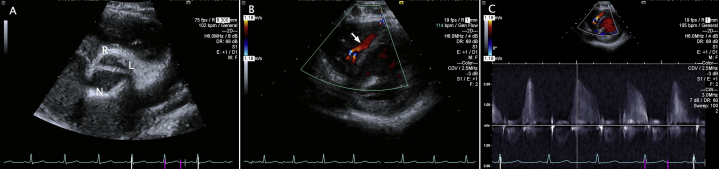
Transthoracic echocardiogram suggestive of coronary anomaly. Panel A demonstrates a BAV with left (L) and right (R) commissural fusion. No LMCA is appreciated arising from the aortic sinus. Panel B demonstrates abnormal linear diastolic color flow signals in the interventricular septum (*arrow*), with confirmatory pulse wave Doppler signals shown in panel C. *N*, noncoronary cusp.

**Figure 2:**
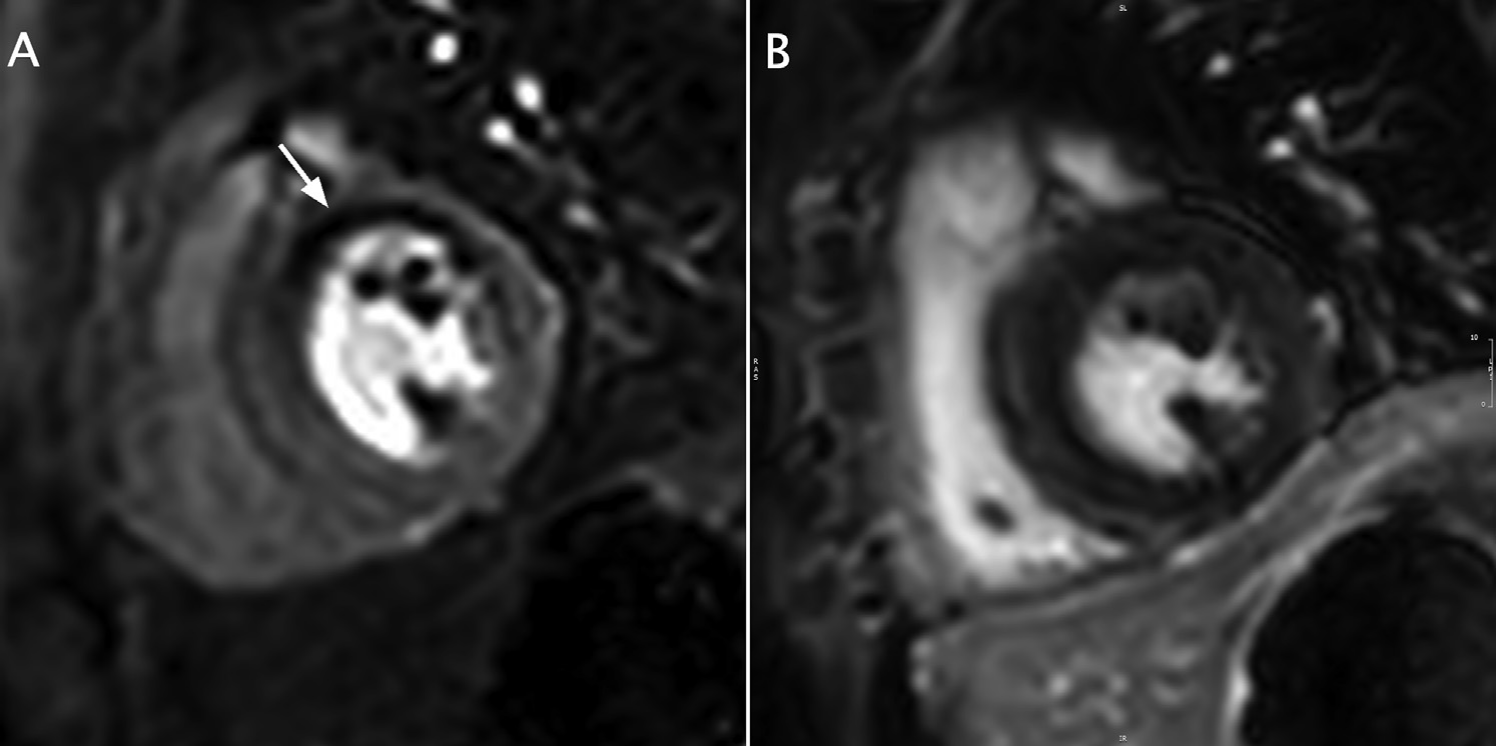
Cardiac magnetic resonance perfusion imaging. **(A)** First-pass perfusion demonstrates a perfusion defect in the mid-anteroseptal and mid-anterior wall (*arrow*). **(B)** No late gadolinium enhancement in the analogous region of myocardium is suggestive of hibernating myocardium.

**Figure 3:**
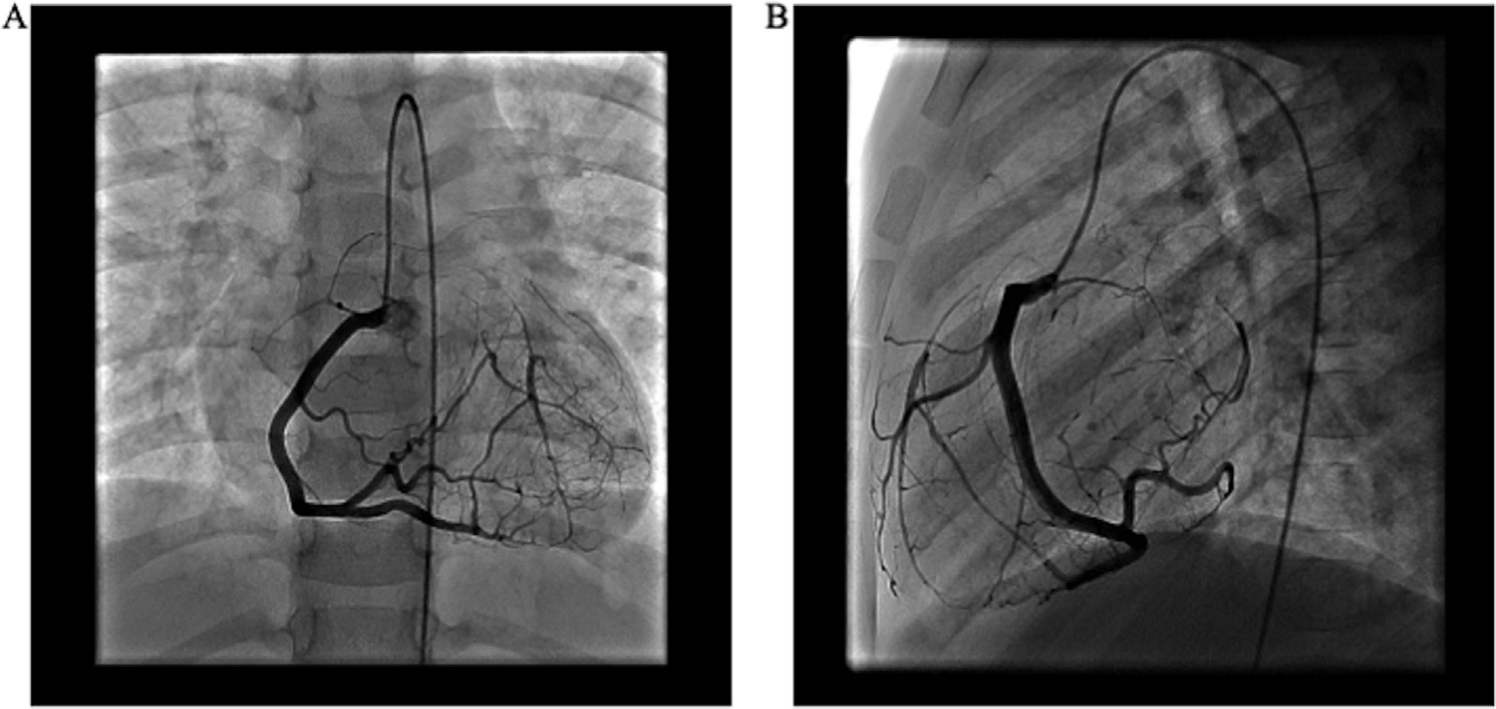
Invasive coronary angiography demonstrates in the anterior-posterior projection **(A)** and lateral projection **(B)** a dilated RCA with retrograde filling of the LAD and circumflex coronary arteries. There is no LMCA segment.

An asymptomatic 3-year-old male was referred for murmur evaluation. Transthoracic echocardiography revealed a bicuspid aortic valve (BAV) with moderate valvar stenosis and regurgitation; the left coronary artery was difficult to visualize. Left ventricular (LV) function was normal. At age 4, he experienced exertional syncope with spontaneous return of consciousness. He was evaluated in a local emergency room and had returned to his baseline with no additional symptoms. He was observed and discharged with no additional testing and instructed to follow up with his cardiologist. Repeat echocardiogram demonstrated stable BAV function, normal biventricular function, and newly appreciated diastolic flow signals in the interventricular septum by color Doppler with continued inability to visualize the left coronary ostium (Figure 1, Videos 1 and 2). The RCA was dilated with normal origin. There were no abnormalities of the mitral valve papillary muscles. Ambulatory rhythm monitor demonstrated no arrhythmias. Cardiac magnetic resonance imaging confirmed RCA dilation with normal origin. The left coronary origin and proximal LMCA were not visualized, although the left anterior descending (LAD) and circumflex arteries were seen in their normal position. Perfusion imaging demonstrated an endocardial perfusion defect in the distribution of the LMCA with no late gadolinium enhancement, consistent with hibernating myocardium (Figure 2). Cardiac catheterization was performed with angiography, demonstrating atresia of the left coronary ostia and long-segment LMCA atresia with retrograde filling of the LAD and circumflex through collaterals from a dilated RCA (Figure 3, Videos 3 and 4). Hemodynamics demonstrated mild aortic valve stenosis (peak gradient, 15 mm Hg), mild LV diastolic dysfunction (LV end-diastolic pressure, 14 mm Hg), and a normal cardiac index of 3.74 L/min/m^2^.

He was referred for surgical coronary revascularization in combination with a Ross-Konno procedure to provide aortic valve competence to prevent coronary hypoperfusion related to aortic regurgitation. Intraoperatively, LMCA atresia was confirmed, which extended beyond the bifurcation of the LAD and circumflex. Given the discontinuity of the circumflex and LAD with a comparably small circumflex artery, a left internal mammary artery (LIMA) to LAD bypass graft was performed.

**Figure 4:**
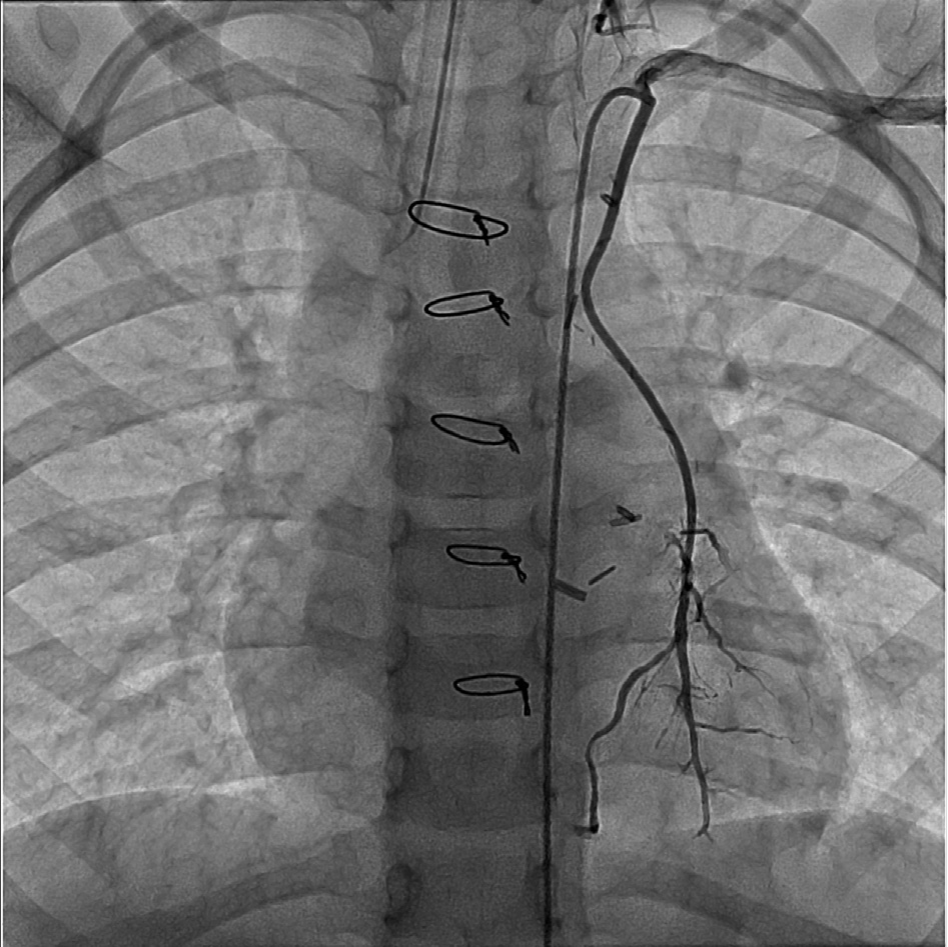
Postoperative angiography demonstrates a widely patent LIMA graft filling the LAD artery.

**Figure 5:**
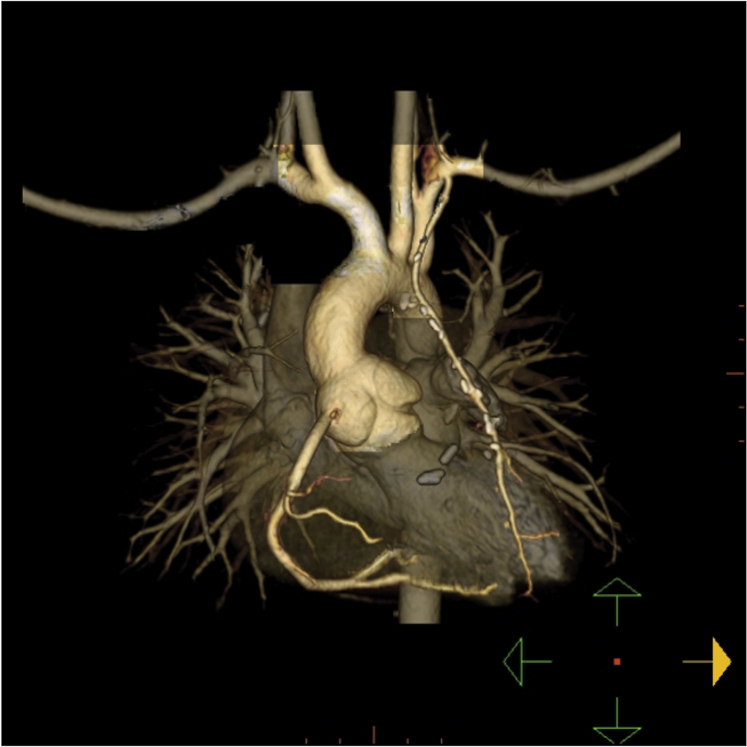
Postoperative cardiac computed tomography angiography with three-dimensional volume-rendered display performed 6 months postoperatively demonstrates a patent LIMA graft filling the LAD artery.

The postoperative course was uneventful without arrhythmias. Postoperative catheterization demonstrated a patent LIMA supplying the LAD (Figure 4, Video 5). Metoprolol was started for cardioprotection and aspirin for antiplatelet effect. He remains without arrhythmias or syncopal episodes and with good activity tolerance on outpatient follow-up. Computed tomography imaging performed 6 months postoperatively demonstrated continued LIMA graft patency (Figure 5).

## Case 2 Presentation

**Figure 6:**
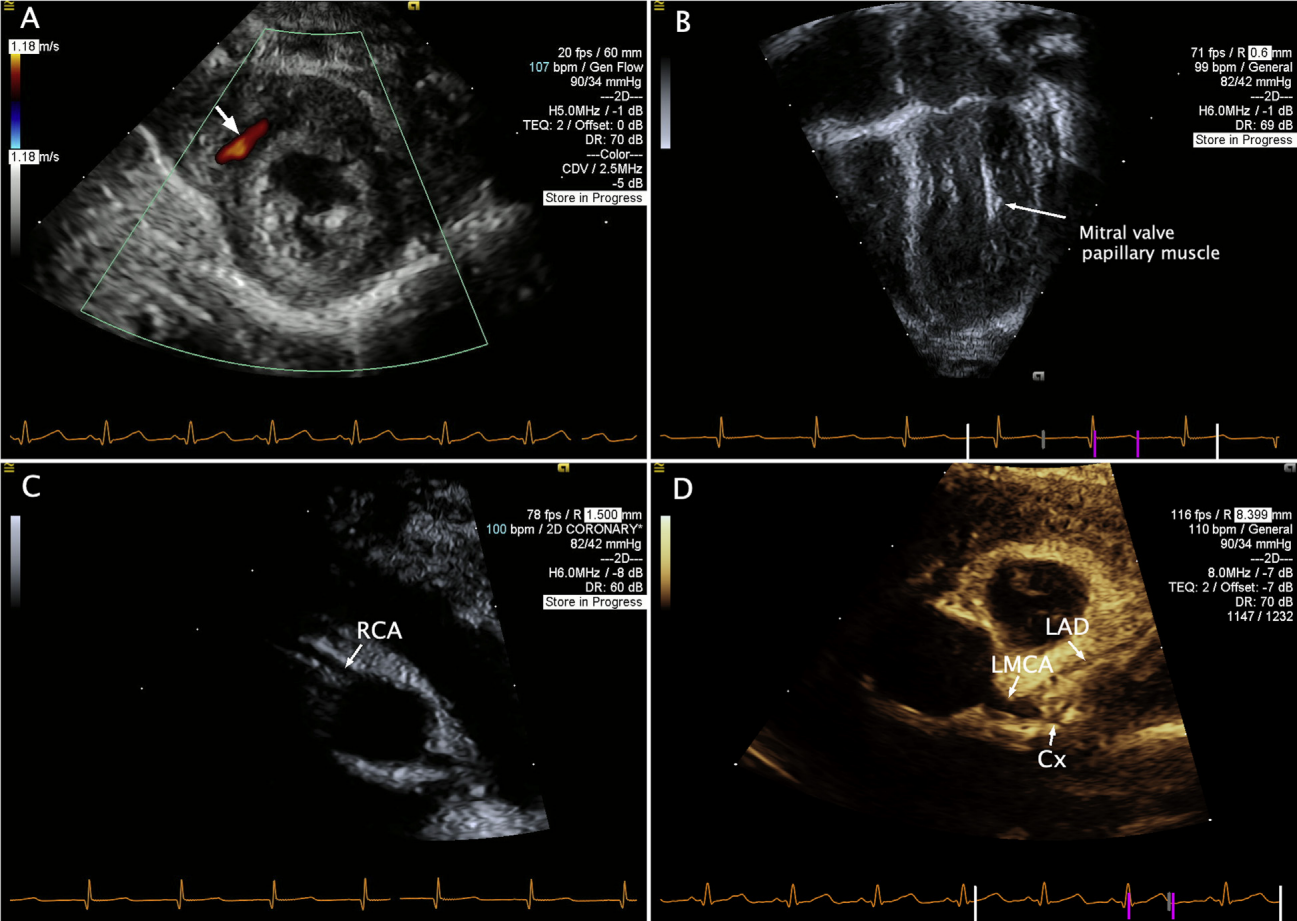
Transthoracic echocardiogram suggestive of coronary anomaly. Panel A demonstrates abnormal diastolic flow signal in the interventricular septum (*arrow*), and panel B shows an abnormally echobright mitral valve papillary muscle, both suspicious for a coronary artery anomaly. Panel C shows a normal RCA origin. Panel D demonstrates a normally positioned LMCA segment with normal branching of the LAD and circumflex (Cx) arteries. However, anterograde color flow could not be demonstrated by color Doppler imaging.

A 6-month-old previously healthy male patient experienced syncope at home. Shortly after feeding, he stiffened before becoming unresponsive, limp, and cyanotic. He was pulseless on emergency medical services arrival. Cardiopulmonary resuscitation was performed, with cardiac rhythm identified as ventricular fibrillation. He was successfully defibrillated. Electrocardiogram was normal. Transthoracic echocardiography demonstrated a structurally normal heart with mild LV systolic dysfunction with ejection fraction of 50%. The mitral valve papillary muscles were abnormally echogenic, and diastolic color Doppler signals were noted in the interventricular septum (Figure 6, Videos 6-8). The LMCA appeared to sit in the normal position, but anterograde flow was not demonstrated by color Doppler. The RCA arose normally. While clinically the patient returned to baseline without further arrhythmias, these findings raised concern for a coronary artery anomaly as the etiology of the ventricular fibrillation arrest.

**Figure 7:**
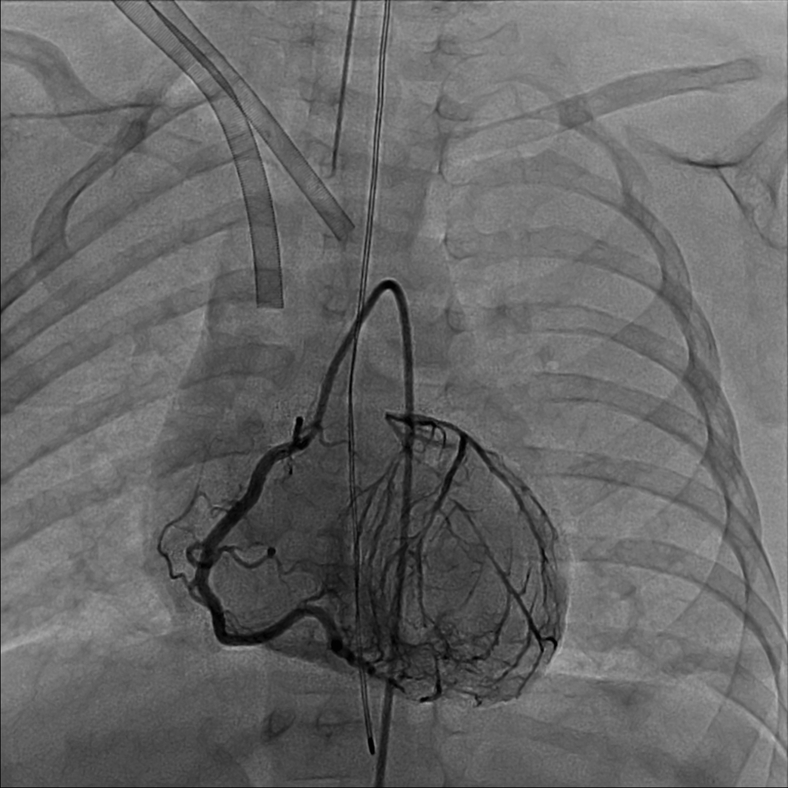
Invasive coronary angiography demonstrates a normally arising RCA with retrograde filling of the left coronary artery system through copious collaterals. The LMCA ends blindly in close proximity to the aortic root.

Diagnostic cardiac catheterization was undertaken. With anesthesia induction, the patient developed hypotension with bradycardic cardiac arrest prompting cardiopulmonary resuscitation with cannulation onto venoarterial extracorporeal membrane oxygenation (ECMO) support. Angiography demonstrated left coronary ostial atresia with retrograde filling of the left coronary artery through collaterals from the RCA, identifying both the LAD and circumflex, which joined to form a well-developed LMCA that ended blindly in proximity to the aortic root (Figure 7, Video 9).

He went to the operating room on ECMO support for coronary artery revascularization. Intraoperative inspection confirmed ostial atresia with a ridge of imperforate tissue in the left aortic sinus. The LMCA was in close proximity to the aortic root, and the left coronary ostia was opened with homograft patch reconstruction of the proximal coronary artery. He weaned from cardiopulmonary bypass without difficulty.

**Figure 8:**
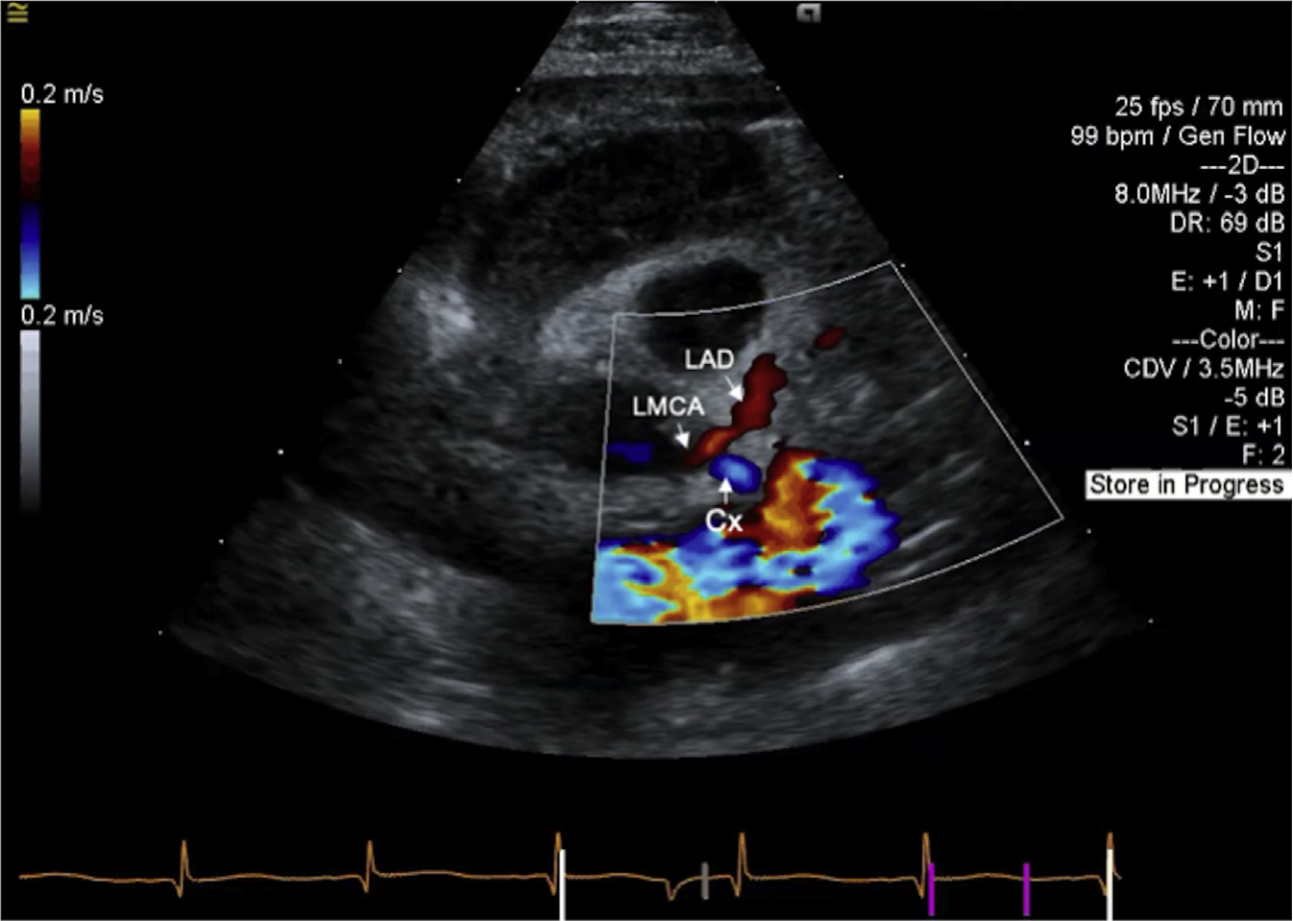
Postoperative transthoracic echocardiogram demonstrates normal antegrade filling of the left coronary artery following opening of the left coronary ostia with homograft patch reconstruction of the proximal coronary artery. *Cx*, Circumflex artery.

Intraoperative angiogram and postoperative echocardiogram demonstrated unobstructed anterograde filling of the left coronary artery (Figure 8, Video 10), and ventricular function normalized. He had no postoperative arrhythmias. Aspirin was started for antiplatelet effect. Following discharge, he has had normal interval growth and development without arrhythmias or cardiovascular symptoms.

## Discussion

Coronary artery anomalies are rare, with congenital ostial atresia of the LMCA being particularly uncommon. Most publications on this topic are case reports and small case series. Alsalehi *et al.*^2^ recently published an elegant review of the literature of a combined 93 cases, 50 of which occurred in the pediatric population. Their work highlights that symptoms are common in this lesion at any age, although pediatric patients are more likely to present with severe symptoms that may include heart failure, syncope, or sudden cardiac death.^2^ Of the pediatric patients, 84.7% underwent surgical coronary revascularization. Importantly, surgery improved outcomes with coronary-associated mortality of 43% in those without surgery compared with 10% in those who did receive coronary revascularization.^2^ Differential diagnosis, particularly in the setting of less severe symptoms, can be broad and may include arrhythmia, seizure, vasovagal syncope, or low cardiac output, which can have varying etiologies including coronary ischemia, myocarditis, or LV dysfunction. As in our patients, echocardiography may be suggestive but typically not diagnostic of LMCA ostial atresia, and a high index of suspicion and close attention to secondary findings suggestive of a coronary anomaly must be maintained to facilitate a definitive diagnosis.

The first case describes a patient with concomitant BAV, which has a previously described association with coronary anomalies, notably with increased prevalence of an absent LMCA segment with separate LAD and circumflex ostia.^3^^,^^4^ There is one prior report of LMCA ostial atresia with concomitant BAV.^5^ Echocardiography in our patient provided diagnostic clues of a left coronary artery anomaly with a dilated RCA, diastolic color Doppler signals in the interventricular septum that represent coronary collateral circulation, and inability to visualize the LMCA.^6^ Catheterization was essential in providing the definitive diagnosis and delineating the coronary anatomy to guide revascularization by LIMA bypass graft. Cardiac magnetic resonance imaging demonstrated viability of the hibernating myocardium in the left coronary distribution.

The second case provides an anatomic contrast, with a well-delineated LMCA in close proximity to the aortic sinus. This allowed for coronary ostial reconstruction for coronary revascularization. Hyperechoic mitral valve papillary muscle, suggestive of ischemia, and diastolic flow signals in the myocardium provided initial diagnostic clues by echocardiography. While catheterization again was critical in providing diagnostic confirmation and anatomic data, our patient's cardiac arrest requiring venoarterial ECMO cannulation with anesthesia highlights the potential hemodynamic fragility of patients with coronary anomalies.

## Conclusion

This case series describes the variable presentation, subtle echocardiographic findings suggestive of coronary atresia, utility of multimodality diagnostic imaging to establish the definitive diagnosis, and surgical management challenges of congenital LMCA ostial atresia in two pediatric patients with favorable outcomes. Additionally, we describe an association of this rare coronary anomaly with BAV, which has only once been previously described.

## Supplementary Data

Video 1 Transthoracic echocardiogram (parasternal short-axis) suggestive of coronary anomaly, demonstrating a bicuspid aortic valve with left (L) and right (R) commissural fusion (*N* = noncoronary cusp). No left main coronary artery is appreciated arising from the aortic sinus. A mildly dilated right coronary artery is appreciated arising from the right aortic sinus.

Video 2 Transthoracic echocardiogram (parasternal right ventricular inflow view) showing an abnormal diastolic flow signal (*a**rrow*) in the interventricular septum.

Video 3 Right coronary angiography demonstrates in the anterior-posterior projection a dilated right coronary artery with retrograde filling of the left anterior descending and circumflex coronary arteries. There is no left main coronary artery segment.

Video 4 Right coronary angiography demonstrates in the lateral projection a dilated right coronary artery with retrograde filling of the left anterior descending and circumflex coronary arteries. There is no left main coronary artery segment.

Video 5 Postoperative angiography in the anterior-posterior projection demonstrates a widely patent left internal mammary artery graft filling the left anterior descending artery.

Video 6 Transthoracic echocardiogram (parasternal short axis) demonstrating a prominent diastolic flow signal in the interventricular septum (*arrow*).

Video 7 Transthoracic echocardiogram (apical 4 chamber) demonstrates the mitral valve papillary muscle is abnormally echobright, which raises suspicion for a coronary artery anomaly.

Video 8 Transthoracic echocardiogram (parasternal short axis) demonstrates a normal right coronary artery (RCA) origin.

Video 9 Right coronary angiography demonstrates a normally arising right coronary artery with retrograde filling of the left coronary artery system through copious collaterals. The left main coronary artery ends blindly in close proximity to the aortic root.

Video 10 Postoperative transthoracic echocardiogram demonstrates normal antegrade filling of the left coronary artery following opening of the left coronary ostia with homograft patch reconstruction of the proximal coronary artery.
